# Design of an embedded inverse-feedforward biomolecular tracking controller for enzymatic reaction processes

**DOI:** 10.1016/j.compchemeng.2017.01.027

**Published:** 2017-04-06

**Authors:** Mathias Foo, Jongrae Kim, Rucha Sawlekar, Declan G. Bates

**Affiliations:** a Warwick Integrative Synthetic Biology Centre, School of Engineering, University of Warwick, Coventry CV4 7AL, UK; b School of Mechanical Engineering, University of Leeds, Leeds LS2 9JT, UK

**Keywords:** CRN, chemical reaction network, DNA, deoxyribonucleic acid, LHS, left-hand-side, RHS, right-hand-side, ODE, ordinary differential equation, PI, proportional-integral, FF, feedforward, IMC, internal model control, DSD, DNA strand displacement, Process control, Enzymatic reaction process, Chemical reaction network theory, Synthetic biology, Biological engineering

## Abstract

Feedback control is widely used in chemical engineering to improve the performance and robustness of chemical processes. Feedback controllers require a ‘subtractor’ that is able to compute the error between the process output and the reference signal. In the case of embedded biomolecular control circuits, subtractors designed using standard chemical reaction network theory can only realise one-sided subtraction, rendering standard controller design approaches inadequate. Here, we show how a biomolecular controller that allows tracking of required changes in the outputs of enzymatic reaction processes can be designed and implemented within the framework of chemical reaction network theory. The controller architecture employs an inversion-based feedforward controller that compensates for the limitations of the one-sided subtractor that generates the error signals for a feedback controller. The proposed approach requires significantly fewer chemical reactions to implement than alternative designs, and should have wide applicability throughout the fields of synthetic biology and biological engineering.

## Highlights

• A new approach for the design of embedded biomolecular controllers is presented.
• The approach circumvents fundamental problems in chemical reaction network theory.
• The control architecture combines inverse-feedforward and feedback control.
• The controller requires fewer chemical reactions for experimental implementation.

## Graphical abstract

**Figure:**
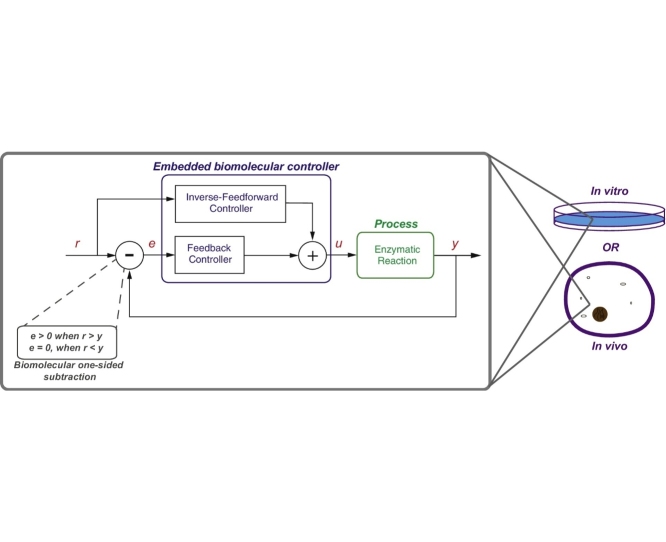

## 1 Introduction

A major challenge in synthetic biology is to develop practically implementable design methods for the synthesis of feedback controllers that achieve reference tracking, i.e. force the output of a biomolecular process of interest to track desired changes in its concentration over time (Hsiao et al., 2015). The design of feedback controllers to control biochemical processes has received significant attention in the literature (see. e.g. Henson, 2003, Baldea et al., 2013), and the construction of synthetic control circuits has become a major focus of research in the new field of synthetic biology. Ideally, such circuits should be made up of well-defined modules consisting only of molecular reactions, in order to allow the realisation of embedded biomolecular control systems (Cosentino et al., 2016). A promising approach to facilitating the design of such circuits is provided by nucleic acid-based chemistry, wherein the design of biomolecular circuits can be done using abstract chemical reaction network (CRN) theory (e.g. Soloveichik et al., 2010), and then translated to deoxyribonucleic acid (DNA) using strand displacement reactions for implementations (Chen et al., 2013). A CRN is a collection of chemical reactions written in the form

(1) $$\underset{\mathrm{Reactants}}{\underset{︸}{X+Y+\ldots }}\overset{\gamma }{\to }\underset{\mathrm{Products}}{\underset{︸}{A+B+\ldots }}$$

where *γ* is the reaction rate, the left-hand-side (LHS) of the reaction consists of reactants and the right-hand-side (RHS) of the reaction consists of products. Most of the chemical reactions considered in this paper are either unimolecular (i.e. one reactant on the LHS of (1)) or bimolecular (i.e. two reactants on the LHS of (1)). According to standard CRN theory (see e.g. Feinberg, 1986, Feinberg, 1988) a CRN with *n* species and *m* reactions can be represented by an ordinary differential equation (ODE) following generalised mass-action kinetic in the form of

$$\frac{dx}{dt}=Pf(x)$$

where $x\in {\mathbb{R}}_{\geq 0}^{n}$ is the species concentration, $f(x)\in {\mathbb{R}}^{m}$ is a function describing the reaction rates of the CRN, $P\in {\mathbb{R}}^{n\times m}$ is the *stoichiometric* matrix that describe the dynamics of the species concentrations following their associated reaction rates, ${\mathbb{R}}_{\geq 0}$ is the non-negative real number set, ℝ is the real number set, and *n* and *m* are positive integers.

**Fig. 1:**
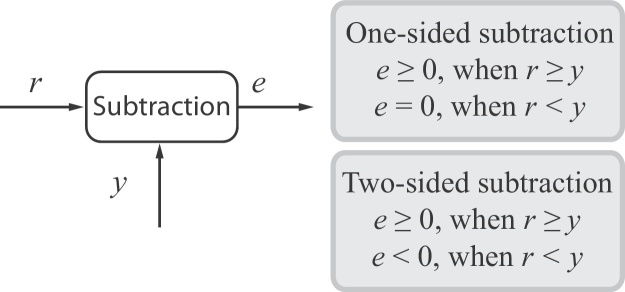
Subtraction operator.

In any reference tracking feedback system, it is imperative that an appropriate error signal can be computed such that the designed controller can take relevant control action to drive the process output towards the intended state. While such a requirement is trivial to satisfy in standard control theory, it is not in the context of CRN theory. This is because a two-sided subtractor (Fig. 1), which is an operator that is able to compute the difference between two input signals regardless of their relative magnitude, is yet to be realised using standard CRN's. For accurate reference tracking, the error *e* should be able to take both positive (*r* > *y*) and negative (*r* < *y*) values. Thus, the aforementioned constraint is a serious impediment to the design of functional biomolecular feedback control systems that will inevitably lead to poor quality reference tracking and potentially even instability.

To the best of the authors' knowledge, almost all previous designs for biomolecular subtractors using CRNs have resulted in only one-sided subtraction. We note that there is a literature on the design of half-subtractors or full-subtractors using digital logic gates realised using CRNs (see e.g. Xu et al., 2013, Lin et al., 2015), however, as our focus is on the design of analog biomolecular circuitry, we exclude this work from our discussion.

The subtraction operator used in our paper is based on the design presented in Buisman et al. (2009), which can be realised using a set of four chemical reactions (see Page 6 of Buisman et al. (2009)). The authors analysed the Jacobian matrix of the ODE associated with the subtraction operator and found that when the resulting subtraction is zero, the fixed point does not exist. Additionally, when the resulting subtraction is negative, the overall system diverges, as the fixed point is unstable. In view of this, the subtraction outputs a positive value when the magnitude of the first component is greater than the second component and zero when the condition is reversed. We further illustrate this point in Section 2.2 of our paper. Some other relevant results on biomolecular subtraction can be found in Salehi et al. (2016) and Song et al. (2016). The design considered in the former used the subtraction operator to realise a biomolecular computation of a Berstein polynomial and their subtraction is equivalent to the design of Buisman et al. (2009). The latter paper proposed frameworks to build operators using DNA strand displacement, and explicitly mentioned that their subtraction operator is one-sided. In Harris et al. (2015), the authors designed a feedback controller for gene expression regulation, which requires the use of a subtraction, but do not propose a detailed biomolecular implementation of the subtraction operator.

An alternative approach to the design of biomolecular subtraction operators can be found in Cosentino et al., 2013, Cosentino et al., 2016 and Bilotta et al., 2015, Bilotta et al., 2016. In this work, the authors developed subtractors that are used to compute the difference between two molecular fluxes, rather than two molecular concentrations. By satisfying certain conditions, and assuming all the reactions involve unitary stoichiometric coefficients with input fluxes constant, the output flux is shown to converge to the difference between the two input fluxes in an asymptotic manner. While the chemical reactions required to realise the subtraction operator in this framework are slightly different to the ones proposed in Buisman et al. (2009), the final ODE representation is exactly the same, and thus also yields a one-sided subtraction.

The only available partial solution to the problem mentioned above is to adopt the design framework proposed in Oishi and Klavins (2011). In this framework, each signal in the biomolecular circuit is implemented as the difference in the concentration of two chemical species. In this way, a two-sided subtraction operator can then be realised. As we show in the following section, however, this approach at least doubles the total number of chemical reactions required to implement the entire feedback circuit. This increase in the number of chemical reactions is highly undesirable as it presents a major challenge for wet lab implementation, and strongly limits the scalability of the design. Moreover, large numbers of chemical reactions potentially increases the probability of unwanted crosstalk interactions. For instance, a circuit whose implementation requires *n* molecular species will increase the potential bimolecular crosstalk interactions by *n*^2^. This has prompted researchers to look into ways to reduce crosstalk, such as requiring a certain number of mismatches for any two distinct recognition domains (see e.g. Qian and Winfree, 2011). Nonetheless, obtaining large numbers of well-behaved sequences with long domains is extremely difficult to achieve in practice.

In this paper, using the available framework for realising one-sided biomolecular subtraction using CRNs (see e.g. Buisman et al., 2009), we propose a design strategy that uses a model-inversion feedforward controller (see e.g. Devasia, 2002, Franklin and Powell, 2014) to circumvent the limitations of the feedback controller when using a one-sided subtraction operator. In addition, our controller design strategy also aims to utilise the minimal number of chemical reactions, to allow for a more scalable and feasible wet lab implementation.

## 2 Materials and methods

### 2.1 One-sided subtraction operator

To the best of authors’ knowledge, all current existing designs for biomolecular subtraction operators that utilise standard CRN theory can only implement one-sided subtraction. A comprehensive list of mathematical operators that can be implemented using CRN's, which includes the one-sided subtraction and its detailed analyses can be found in Buisman et al. (2009). Following the design of Buisman et al. (2009), the subtraction operator can be realised using the following abstract chemical reactions:

(2) $$\begin{array}{cc} x_{i,1}\overset{\gamma }{\to }x_{i,1}+x_{o}, & x_{itd}+x_{o}\overset{\gamma }{\to }\emptyset \\ x_{i,2}\overset{\gamma }{\to }x_{i,2}+x_{itd}, & x_{o}\overset{\gamma }{\to }\emptyset \end{array}$$

where *x*_*i*,1_ and *x*_*i*,2_ are the two inputs, *x*_*o*_ is the resulting output, *x*_*itd*_ is the intermediate states and *γ* is the reaction rate. Note that this one-sided subtraction operator is realised using *four* abstract chemical reactions. Using generalised mass-action kinetics (see e.g. Feinberg, 1986), these abstract chemical reactions can be represented by ODE's, where the corresponding ODE's for (2) are given by

(3) $$\begin{array}{c} \frac{dx_{o}}{dt}=\gamma (x_{i,1}-x_{o}x_{itd}-x_{o})\\ \frac{dx_{itd}}{dt}=\gamma (x_{i,2}-x_{o}x_{itd}) \end{array}$$

At steady state, *x*_*i*,2_ = *x*_*o*_*x*_*itd*_, leading to *x*_*o*_ = *x*_*i*,1_ − *x*_*i*,2_. By analysing the Jacobian matrix of the ODE's relating to this subtraction operator, it has been shown in Buisman et al. (2009) that when the subtraction of two components results in either zero or a negative value, the fixed point does not exist or the system converges to an unstable fixed point, respectively. Hence, *x*_*o*_ = 0 when *x*_*i*,1_ < *x*_*i*,2_ and *x*_*o*_ = *x*_*i*,1_ − *x*_*i*,2_ > 0 when *x*_*i*,1_ ≥ *x*_*i*,2_, making the subtraction one-sided. Other example of one-sided subtraction operators can be found in Cosentino et al. (2016) – in these cases the operators are used to compute the difference of molecular fluxes, rather than concentrations.

### 2.2 Two-sided subtraction operator

As mentioned in Section 1, the only known framework to date that offers a partial solution to the realisation of a two-sided subtraction operator can be found in Oishi and Klavins (2011). In that proposed framework, a signal, *u* is represented as a difference between two chemical species i.e. *u* : = *u*^+^ − *u*^−^. This representation results in the chemical species having positive and negative components. To illustrate how such representation can achieve a two-sided subtraction, we consider first how the summation operator can be realised using abstract chemical reactions. The abstract chemical reactions for the summation operator are given by

(4) $$\begin{array}{c} x_{i,1}^{+}\overset{\gamma }{\to }x_{i,1}^{+}+x_{o}^{+},\;x_{i,1}^{-}\overset{\gamma }{\to }x_{i,1}^{-}+x_{o}^{-},\;x_{i,1}^{+}+x_{i,1}^{-}\overset{\eta }{\to }\emptyset \\ x_{i,2}^{+}\overset{\gamma }{\to }x_{i,2}^{+}+x_{o}^{+},\;x_{i,2}^{-}\overset{\gamma }{\to }x_{i,2}^{-}+x_{o}^{-},\;x_{i,2}^{+}+x_{i,2}^{-}\overset{\eta }{\to }\emptyset \\ x_{o}^{+}\overset{\gamma }{\to }\emptyset ,\;x_{o}^{-}\overset{\gamma }{\to }\emptyset ,\;x_{o}^{+}+x_{o}^{-}\overset{\eta }{\to }\emptyset \end{array}$$

where *η* is a reaction rate such that *η* ≫ *γ*. The corresponding ODEs are given by

(5) $$\begin{array}{c} \frac{dx_{o}^{+}}{dt}=\gamma (x_{i,1}^{+}+x_{i,2}^{+}-x_{o}^{+})-\eta x_{o}^{+}x_{o}^{-}\\ \frac{dx_{o}^{-}}{dt}=\gamma (x_{i,1}^{-}+x_{i,2}^{-}-x_{o}^{-})-\eta x_{o}^{+}x_{o}^{-}\\ \frac{dx_{o}}{dt}=\frac{dx_{o}^{+}}{dt}-\frac{dx_{o}^{-}}{dt}=\gamma (x_{i,1}+x_{i,2}-x_{o}) \end{array}$$

where at steady state (i.e. *dx*_*o*_/*dt* = 0), *x*_*o*_ = *x*_*i*,1_ + *x*_*i*,2_.

Now, for the subtraction operator, its abstract chemical reactions are given by

(6) $$\begin{array}{c} x_{i,1}^{+}\overset{\gamma }{\to }x_{i,1}^{+}+x_{o}^{+},\;x_{i,1}^{-}\overset{\gamma }{\to }x_{i,1}^{-}+x_{o}^{-},\;x_{i,1}^{+}+x_{i,1}^{-}\overset{\eta }{\to }\emptyset \\ x_{i,2}^{+}\overset{\gamma }{\to }x_{i,2}^{+}+x_{o}^{-},\;x_{i,2}^{-}\overset{\gamma }{\to }x_{i,2}^{-}+x_{o}^{+},\;x_{i,2}^{+}+x_{i,2}^{-}\overset{\eta }{\to }\emptyset \\ x_{o}^{+}\overset{\gamma }{\to }\emptyset ,\;x_{o}^{-}\overset{\gamma }{\to }\emptyset ,\;x_{o}^{+}+x_{o}^{-}\to \eta \emptyset \end{array}$$

Notice the difference of the superscripts + and − in the abstract chemical reaction compared to (4). The corresponding ODEs are given by

(7) $$\begin{array}{c} \frac{dx_{o}^{+}}{dt}=\gamma (x_{i,1}^{+}+x_{i,2}^{-}-x_{o}^{+})-\eta x_{o}^{+}x_{o}^{-}\\ \frac{dx_{o}^{-}}{dt}=\gamma (x_{i,1}^{-}+x_{i,2}^{+}-x_{o}^{-})-\eta x_{o}^{+}x_{o}^{-}\\ \frac{dx_{o}}{dt}=\frac{dx_{o}^{+}}{dt}-\frac{dx_{o}^{-}}{dt}=\gamma (x_{i,1}-x_{i,2}-x_{o}) \end{array}$$

where at steady state, *x*_*o*_ = *x*_*i*,1_ − *x*_*i*,2_. Because the signal is represented as the difference of positive and negative components, *x*_*o*_ can be properly computed regardless of the relative magnitude of *x*_*i*,1_ and *x*_*i*,2_. Note that both the summation and subtraction operators in (4), (6) are realised using *nine* abstract chemical reactions.

Now, note that the summation of two concentrations that is equivalent to (5) with positive signals could also have been realised by employing the following three abstract chemical reactions (Buisman et al., 2009): $x_{i,1}\overset{\gamma }{\to }x_{i,1}+x_{o}$, $x_{i,2}\overset{\gamma }{\to }x_{i,2}+x_{o}$ and $x_{o}\overset{\gamma }{\to }\emptyset$. This realisation does not require the signal to be represented using positive/negative components. Surprisingly, however, an equivalent way of representing the subtraction operator as in (7) seems not to exist, as there are no associated abstract chemical reactions to realise it. We shall further demonstrate this point below.

Consider the following two reactions: $x_{i,1}\overset{\gamma }{\to }x_{i,1}+y$ and $y\overset{\gamma }{\to }\emptyset$. Their corresponding ODEs are *dy*/*dt* =+ *γx*_*i*,1_ and *dy*/*dt* =− *γy* respectively. Their final ODE expression can then be obtained by summing these two equations together, i.e. *dy*/*dt* = *γ*(*x*_*i*,1_ − *y*). Now, for the subtraction operator, the corresponding ODE is given by *dy*/*dt* = *γ*(*x*_*i*,1_ − *x*_*i*,2_ − *y*). We have already shown how *dy*/*dt* = *γ*(*x*_*i*,1_ − *y*) can be obtained. Therefore, we simply need another abstract chemical reaction that can achieve *dy*/*dt* =− *γx*_*i*,2_. With the sign on the RHS of the ODE being negative, one would expect to write *y* on the LHS of the abstract chemical reaction. In addition, we require the multiplication of *x*_*i*,2_ with *γ*, which means *x*_*i*,2_ has to be on the LHS of the abstract chemical reaction as well. A natural first attempt would then be to write $x_{i,2}+y\overset{\gamma }{\to }\emptyset$. However, a sum of reactants in an abstract chemical reaction leads to multiplication in the corresponding ODE, i.e. *dy*/*dt* =− *γx*_*i*,2_*y*. If we are to move *y* to the RHS of the abstract chemical reaction, i.e. $x_{i,2}\overset{\gamma }{\to }x_{i,2}+y$, its corresponding ODE would then be *dy*/*dt* =+ *γx*_*i*,2_. Thus, there is no way to realise *dy*/*dt* =− *γx*_*i*,2_ using standard abstract chemical reactions. This is the reason why the positive/negative components formalism introduced by Oishi and Klavins (2011) is needed to realise a two-sided subtraction operator.

While the formalism proposed in Oishi and Klavins (2011) is able to realise two-sided subtraction, it also inevitably leads to a large increase in the number of abstract chemical reactions required, since it must be used to realise *all* operators and components in the circuit, even though in many cases there are simpler alternatives available (e.g. the summation operator). From the point of view of experimental implementation, it is highly desirable to keep the number of abstract chemical reactions required as small as possible, thus motivating our attempt to formulate a design strategy that can cope with the limitations of a one-sided subtraction operator and thus allow us to avoid using positive/negative components.

### 2.3 Enzymatic reaction processes

In this paper, we focus on the problem of controlling enzymatic reaction processes. Such processes are ubiquitous in cell biology, with some notable examples including protein phosphorylation by kinases, metabolic synthesis pathways and anaerobic fermentation of glucose to ethanol in yeast (see e.g. Segel, 1975, Galazzo and Bailey, 1990, Heinrich et al., 2002, Faeder et al., 2003, Hatakeyama et al., 2003, Charusanti et al., 2004, Gershon and Shaked, 2008).

The main function of enzymes is to act as the catalysts of biochemical systems (see e.g. Taylor et al., 2008). Disruption to the regulation of enzymatic reaction processes can therefore have significant adverse effect on biological function, and the ability to accurately control the dynamics of enzymatic reaction processes at their optimal levels is crucial to a wide range of natural and engineered biochemical systems.

The chemical reactions describing the enzymatic reaction process in its simplest form (see e.g. Segel, 1975, Gershon and Shaked, 2008) are given by

(8) $$\begin{array}{c} x_{in}+x_{e}\overset{k_{r1}}{\to }x_{i}\\ x_{i}\overset{k_{r2}}{\to }x_{out}+x_{e}\\ x_{out}\overset{k_{r3}}{\to }\emptyset \end{array}$$

where *k*_*r*1_, *k*_*r*2_ and *k*_*r*3_ are the process binding, catalytic and degradation rates respectively. *x*_*in*_, *x*_*e*_, *x*_*i*_ and *x*_*out*_ usually represent the substrate, enzyme, enzyme-substrate and product respectively. In a typical enzymatic reaction, the enzyme combines with its specific substrate to form the enzyme-substrate complex. After the reaction, this enzyme–substrate complex breaks up resulting in the associated product and the enzyme itself. This enzyme is unchanged and once separated from the complex is free to interact again with more substrate.

The corresponding ODE's for (8) are then given by

(9) $$\begin{array}{c} \frac{dx_{i}}{dt}=k_{r1}x_{in}x_{e}-k_{r2}x_{i}\\ \frac{dx_{out}}{dt}=k_{r2}x_{i}-k_{r3}x_{out} \end{array}$$

Here, we assume that the total concentration of *x*_*e*_ and *x*_*i*_ represented as *x*_*T*_ = *x*_*e*_ + *x*_*i*_ is constant.

## 3 Theory/calculation

### 3.1 Derivation of inverse-feedforward controller

The basic aim of the inverse-feedforward controller is to invert the relevant dynamics of the process at steady state in order to provide accurate steady-state tracking of reference signals. From (9), at steady-state, (i.e. setting both *dx*_*i*_/*dt* and *dx*_*out*_/*dt* to zero), we have

(10) $$\begin{array}{c} k_{r1}x_{in}x_{e}=k_{r2}x_{i}\\ k_{r2}x_{i}=k_{r3}x_{out} \end{array}$$

After some algebraic manipulation, we get

(11) $$x_{in}=\frac{\alpha x_{out}}{\left(\beta -\delta x_{out}\right)}$$

where *α* = *k*_*r*2_*k*_*r*3_, *β* = *k*_*r*1_*k*_*r*2_*x*_*T*_ and *δ* = *k*_*r*1_*k*_*r*3_. For reference tracking, the steady state value of the output, *x*_*out*_ should reach the reference signal, *r*. By substituting *x*_*out*_ = *r* and rewriting *x*_*in*_ = *u* into (11), we obtain the required control input signal such that the process is able to track the reference signal at steady state, which is given by

(12) $$u=\frac{\alpha r}{\left(\beta -\delta r\right)}$$

**Fig. 2:**
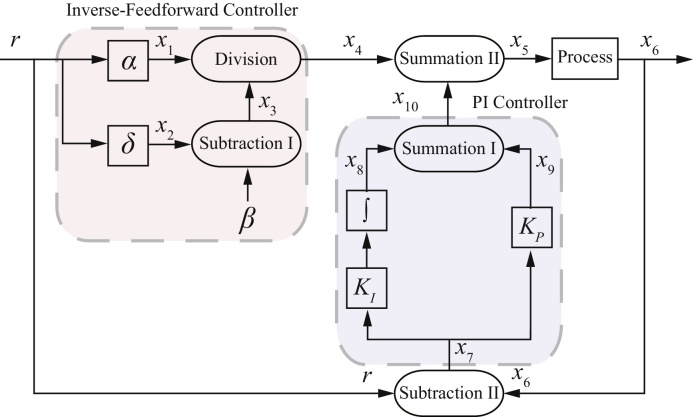
Block diagram configuration of the proposed tracking controller.

The control signal given in (12) essentially amounts to an open loop control strategy, in which the relevant dynamics of the process are inverted in order to achieve perfect steady-state tracking of reference signals. In practice, of course, the inversion will not be exact, due to model uncertainty, and there is also no way to control the transient dynamics of the closed loop system using this approach, which could lead to the presence of steady state errors. To address these limitations, the feedforward controller is used together with a classical proportional-integral (PI) feedback controller, as shown in Fig. 2. The main purpose of the feedback controller is to correct for errors introduced by model mismatch and to control the transient behaviour of the process. Thus, the final control signal acting on the process is given by the sum of the outputs of the feedforward and feedback controllers. Note that Subtraction I and Subtraction II shown in Fig. 2 are both one-sided subtraction operators.

### 3.2 Abstract chemical reaction representation of the tracking controller

Here, we show how the tracking controller may be realised using abstract chemical reactions. As shown in Fig. 2, the feedforward controller requires two gain operators, one division operator and one (one-sided) subtraction operator. Following the design procedure for all those operators given in Buisman et al. (2009), the associated abstract chemical reactions for the feedforward controller part are given as follows:

[Gain, *α*:]

(13) $$\begin{array}{c} r\overset{\alpha \gamma_{G_{\alpha }}}{\to }r+x_{1}\\ r\overset{\gamma_{G_{\alpha }}}{\to }\emptyset \end{array}$$

where $\gamma_{G_{\alpha }}$ is *α* gain reaction rate.

[Gain, *δ*:]

(14) $$\begin{array}{c} r\overset{\delta \gamma_{G_{\delta }}}{\to }r+x_{2}\\ r\overset{\gamma_{G_{\delta }}}{\to }\emptyset \end{array}$$

where $\gamma_{G_{\delta }}$ is the *δ* gain reaction rate.

[Subtraction I:]

(15) $$\begin{array}{c} \beta \overset{\gamma_{Sb_{I}}}{\to }\beta +x_{3}\\ x_{2}\overset{\gamma_{Sb_{I}}}{\to }x_{2}+x_{s}\\ x_{3}\overset{\gamma_{Sb_{I}}}{\to }\emptyset \\ x_{3}+x_{s}\overset{\gamma_{Sb_{I}}}{\to }\emptyset \end{array}$$

where $\gamma_{Sb_{I}}$ is Subtraction I reaction rate and *x*_*s*_ is the intermediate species involved in Subtraction I.

[Division:]

(16) $$\begin{array}{c} x_{1}\overset{\gamma_{D}}{\to }x_{1}+x_{4}\\ x_{3}+x_{4}\overset{\gamma_{D}}{\to }x_{3} \end{array}$$

where *γ*_*D*_ is the division reaction rate.

For the PI feedback controller, the associated abstract chemical reactions are given as follows:

[Integral gain:]

(17) $$x_{7}\overset{K_{I}}{\to }x_{7}+x_{8}$$

where *K*_*I*_ is the integral gain.

[Proportional gain:]

(18) $$\begin{array}{c} x_{7}\overset{\gamma_{G_{K_{P}}}K_{P}}{\to }x_{7}+x_{9}\\ x_{9}\overset{\gamma_{G_{K_{P}}}}{\to }\emptyset \end{array}$$

where *K*_*P*_ is the proportional gain and $\gamma_{G_{K_{P}}}$ is the gain reaction rates.

[Summation I:]

(19) $$\begin{array}{c} x_{8}\overset{\gamma_{Sm_{I}}}{\to }x_{8}+x_{10}\\ x_{9}\overset{\gamma_{Sm_{I}}}{\to }x_{9}+x_{10}\\ x_{10}\overset{\gamma_{Sm_{I}}}{\to }\emptyset \end{array}$$

where $\gamma_{Sm_{I}}$ is Summation I reaction rate.

The error resulting from model mismatch and transient effects is computed by Subtraction II, where the corresponding abstract chemical reactions are given by

[Subtraction II:]

(20) $$\begin{array}{c} r\overset{\gamma_{Sb_{\mathrm{II}}}}{\to }r+x_{7}\\ x_{6}\overset{\gamma_{Sb_{\mathrm{II}}}}{\to }x_{6}+x_{t}\\ x_{7}\overset{\gamma_{Sb_{\mathrm{II}}}}{\to }\emptyset \\ x_{t}+x_{7}\overset{\gamma_{Sb_{\mathrm{II}}}}{\to }\emptyset \end{array}$$

where $\gamma_{Sb_{\mathrm{II}}}$ is Subtraction II reaction rate and *x*_*t*_ is the intermediate species involved in Subtraction II.

The overall control signal to be applied to the process is the summation of the control signals from both the feedforward and feedback controllers, where the abstract chemical reactions are given by

[Summation II:]

(21) $$\begin{array}{c} x_{4}\overset{\gamma_{Sm_{\mathrm{II}}}}{\to }x_{4}+x_{5}\\ x_{10}\overset{\gamma_{Sm_{\mathrm{II}}}}{\to }x_{10}+x_{5}\\ x_{5}\overset{\gamma_{Sm_{\mathrm{II}}}}{\to }\emptyset \end{array}$$

where $\gamma_{Sm_{\mathrm{II}}}$ is Summation II reaction rate.

With the control signal, *x*_5_ as the input to the process, the abstract chemical reactions for the process to be controlled are given by (8) with *x*_*in*_ = *x*_5_ and *x*_*out*_ = *x*_6_. Thus, the number of chemical reactions needed to realise the complete control circuit is 26. On the other hand, if the design framework that utilises two-sided subtraction as proposed in Oishi and Klavins (2011) is to be used to design just a standard PI feedback controller, 36 chemical reactions would be required in the circuit, an increase in complexity of 28%.

### 3.3 Ordinary differential equation representation of the tracking controller

Using generalised mass-action kinetics, the corresponding ODE's for all the abstract chemical reactions described in Section 3.2 are given by

[Feedforward controller:]

(22) $$\begin{array}{c} \frac{dx_{1}}{dt}=\gamma_{G_{\alpha }}(\alpha r-x_{1})\\ \frac{dx_{2}}{dt}=\gamma_{G_{\delta }}(\delta r-x_{2})\\ \frac{dx_{3}}{dt}=\gamma_{Sb_{I}}(\beta -x_{s}x_{3}-x_{3})\\ \frac{dx_{s}}{dt}=\gamma_{Sb_{I}}(x_{2}-x_{s}x_{3})\\ \frac{dx_{4}}{dt}=\gamma_{D}(x_{1}-x_{3}x_{4}) \end{array}$$

[Feedback controller:]

(23) $$\begin{array}{c} \frac{dx_{8}}{dt}=K_{I}x_{7}\\ \frac{dx_{9}}{dt}=\gamma_{G_{K_{P}}}(K_{P}x_{7}-x_{9})\\ \frac{dx_{10}}{dt}=\gamma_{Sm_{I}}(x_{8}+x_{9}-x_{10}) \end{array}$$

[Subtraction II:]

(24) $$\begin{array}{c} \frac{dx_{7}}{dt}=\gamma_{Sb_{\mathrm{II}}}(r-x_{t}x_{7}-x_{7})\\ \frac{dx_{t}}{dt}=\gamma_{Sb_{\mathrm{II}}}(x_{6}-x_{t}x_{7}) \end{array}$$

[Summation II:]

(25) $$\frac{dx_{5}}{dt}=\gamma_{Sm_{\mathrm{II}}}(x_{4}+x_{10}-x_{5})$$

[Process:]

(26) $$\begin{array}{c} \frac{dx_{i}}{dt}=k_{r1}x_{5}x_{e}-k_{r2}x_{i}\\ \frac{dx_{6}}{dt}=k_{r2}x_{i}-k_{r3}x_{6} \end{array}$$

with *x*_*T*_ = *x*_*e*_ + *x*_*i*_ is constant.

## 4 Results and discussion

### 4.1 Limitation of feedback control with one-sided subtraction

**Fig. 3:**
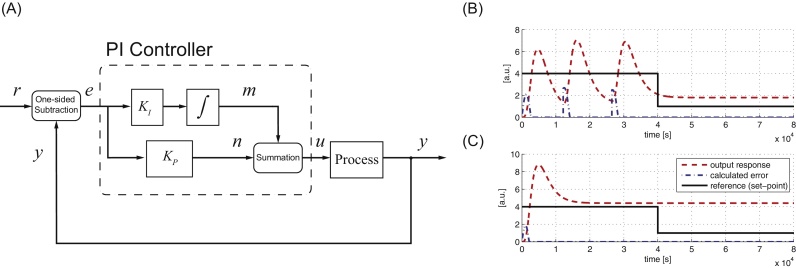
(A) Block diagram configuration of a standard closed-loop feedback control system using a PI controller with a one-sided subtraction operator. (B) System response with PI feedback controller and one-sided subtraction operator with lower control gain, (C) with higher control gain.

In this section, we first illustrate the effect of using a one-sided subtraction operator when a standard tracking feedback controller is used. Fig. 3(A) shows the configuration of a standard feedback system with a PI controller using a one-sided subtraction operator and the PI controller is tuned using the standard Ziegler–Nichol or Internal Model Control (IMC) methods (see e.g. Ogata, 2010, Rivera et al., 1986). From Fig. 3(B), at time 0 to 40,000s, the feedback controller attempts to track the reference value but as overshoot occurs, we have the situation that the reference value is smaller than the output value resulting in no control action before the undershoot where the situation is reversed. This leads to the oscillatory behaviour observed within that time span. At time 40,000s to 80,000s, the reference value is stepped down and we have the situation of the reference value being smaller than the output value. This means the error given by the one-sided subtraction is always zero and the PI controller exerts no control action, resulting in the large steady state error. The performance does not improve despite repeated attempts to tune the PI controller gain – one of the best tunings is shown in Fig. 3(C), where the PI controller is still unable to achieve proper reference tracking.

### 4.2 Inverse-feedforward controller with one-sided subtraction

**Fig. 4:**
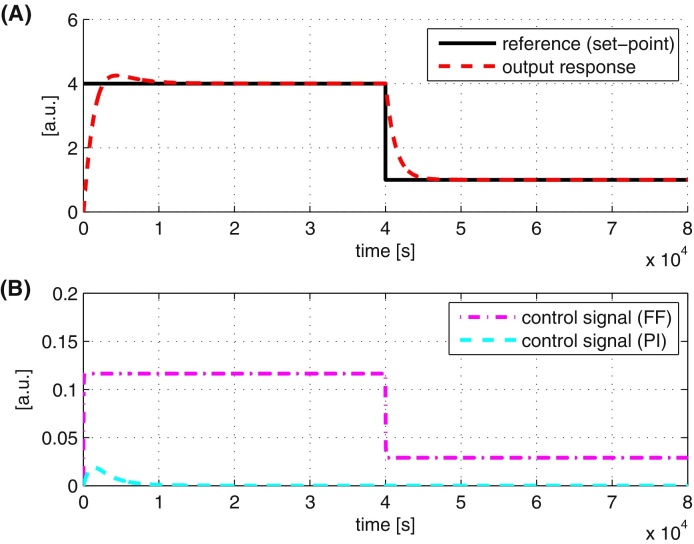
System responses with the tracking controller. (A) Output and reference signals. (B) Control signals from inverse-feedforward (FF) and PI controllers.

In Fig. 4, we show the results of repeating the above simulation with our inverse-feedforward controller architecture from Fig. 2. All the relevant parameters used in the simulation are summarised in Table 1. All units are assumed to be defined appropriately. The performance of the controller is good as it is able to track the reference signal properly. The contribution of the two controllers is as expected, where most of the control action is given by the feedforward controller while the PI controller is operative when dealing with transients. The parameters of the PI controller are obtained using standard tuning methods e.g., Ziegler–Nichol/IMC method (see e.g. Ogata, 2010, Rivera et al., 1986) followed by further fine tuning based on closed-loop simulations.

### 4.3 Robustness and sensitivity analysis

Here, the robustness of the controller when implemented in closed-loop is investigated using Monte Carlo simulations, where all the parameters in (22), (23), (24), (25), (26) are randomly drawn from a uniform distribution and the above simulations are repeated. The number of Monte Carlo simulations required to obtain various levels of estimation uncertainty with known probability are determined following Chernoff bound (Vidyasagar, 1998). Following the guidelines given in Williams (2001), a total number of 1060 simulations are required to accomplish an accuracy level of 0.05 with confidence level of 99% (Vidyasagar, 1998, Menon et al., 2009). All the parameters are varied within ranges of 10% around their nominal values. Mathematically, this is written as *θ*(1 + 0.1*Δ*), where $\theta \in \{\gamma_{G_{\alpha },i},\gamma_{G_{\delta },i},\gamma_{Sb_{I},j},\gamma_{D,i},\gamma_{G_{K_{P}},i},\gamma_{Sm_{I},k},\gamma_{Sb_{\mathrm{II}},j},\gamma_{Sm_{\mathrm{II}},k}$, *K*_*P*_, *K*_*I*_, *k*_*r*1_, *k*_*r*2_, *k*_*r*3_}, where *i* ∈ {1, 2}, *j* ∈ {1, 2, 3, 4, 5} and *k* ∈ {1, 2, 3}. *Δ* is a random number from the uniform distribution in [−1,1]. Note that all the associated reaction rates are split according to the number of chemical reactions in which they are involved.

**Fig. 5:**
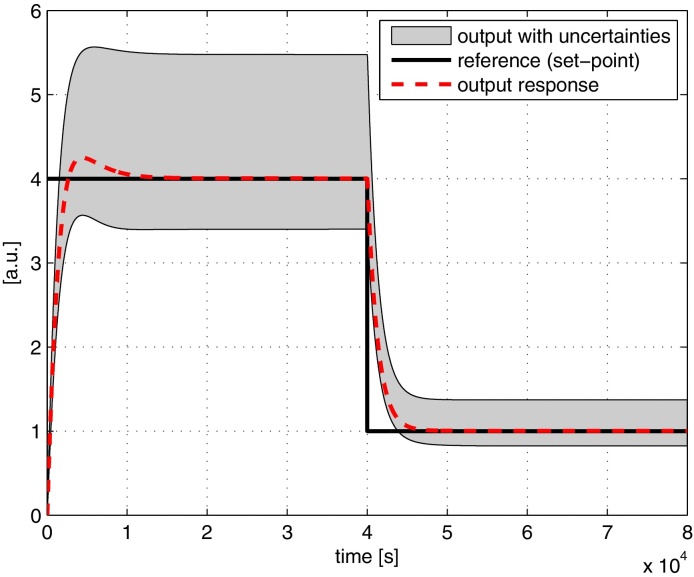
Robustness analysis of the tracking controller.

The simulation results are shown in Fig. 5. The region shaded in grey is the output envelope encompassing all responses from 1060 Monte Carlo simulations for randomly perturbed parameters in the range of ±10% from the nominal values. The controller displays a good level of robust performance with no stability issues as a result of varying parameters. The simulation was repeated with perturbations up to ±100% (i.e. *θ*(1 + *Δ*) with *Δ* = 1) and no instability was observed.

Despite the robustness analysis showing good performance with no stability issues, one notable result warranting further investigation is the presence of steady state error. It is known that any controller with integral action should eliminate steady state errors even in the presence of model uncertainty and this is not observed in our simulation. This is due to the effect of uncertainty on the biomolecular subtraction and summation operators. From (22), (23), (24), (25), (26), to achieve exact summation and subtraction, their associated reaction rates (*γ*_*Sb*_ and *γ*_*Sm*_) are required to be identical. This can be seen by considering the following summation, i.e., *dy*/*dt* = *γ*_1_*x*_1_ + *γ*_2_*x*_2_ − *γ*_3_*y*. The summation is exact when *γ*_1_ = *γ*_2_ = *γ*_3_, where at steady state, *y* = *x*_1_ + *x*_2_. When the parameters are perturbed by uncertainty, however, this results in the summation reaction rates no longer being identical (*γ*_1_ ≠ *γ*_2_ ≠ *γ*_3_). Then, at steady state, *y* = (*γ*_1_/*γ*_3_)*x*_1_ + (*γ*_2_/*γ*_3_)*x*_2_, and the summation is no longer exact. Thus, these non-proper summation and subtraction operators are likely to result in the controller being unable to compute the correct control signal in response to the error, leading to the observed steady state error. To confirm this, we performed parameter sensitivity analysis, whereby each of the parameters shown in Table 1 are multiplied by a factor that ranges from 0.5 to 2.0 with increments of 0.1. Sensitivity is quantified by computing the relative steady state error at times 40,000s (step-up) and 80,000s (step-down) or mathematically,

(27) $$\begin{array}{c} \mathrm{Relative}\;e_{\mathrm{ss},U}=\frac{\hat{y}(40,000)-4}{\hat{y}(40,000)}\\ \mathrm{Relative}\;e_{\mathrm{ss},D}=\frac{\hat{y}(80,000)-1}{\hat{y}(80,000)} \end{array}$$

where $\hat{y}$ is the output response subjected to parameter sensitivity. Table 2 shows the maximum relative steady state errors resulting from tracking the step-up and step-down of the reference signal.

The results of the sensitivity analysis confirm that the steady state error is largely attributable to the Summation II and Subtraction II modules. These two operators are responsible for computing the total control signal to the process and the error to the PI controller respectively. The parameters associated with the feedforward controller are also highly sensitive. This is expected given that the feedforward controller operates by inverting the relevant dynamics of the process at steady state. Thus, any changes to the parameters within the feedforward controller could result in the computation of the incorrect control signal to negate the process dynamic, which subsequently leads to large steady state error. Care is thus required when specifying and implementing the reaction rates related to the controller and the summation and subtraction operators.

### 4.4 Simplified feedforward controller

The realisation of the inverse-feedforward controller involves a total of 26 abstract chemical reactions. While this produces a reduction of circuit complexity by 28% compared to using the framework proposed in Oishi and Klavins (2011), further reductions to the number of abstract chemical reactions would be highly desirable. This can be achieved using an alternative way to realise this feedforward controller, as follows. From (12), which we rewrite here,

$$u=\frac{\alpha r}{\left(\beta -\delta r\right)}$$

we note that *r* appears in both the numerator and denominator. This fractional representation can often be approximated by a polynomial to obtain a simplified representation. Taking the Taylor series expansion of *u* at r^*^ = 0, we have the following:

(28) $$\frac{\alpha r}{\left(\beta -\delta r\right)}\approx \frac{\alpha r^{*}}{\left(\beta -\delta r^{*}\right)}+\frac{\alpha \beta }{{\left(\beta -\delta r^{*}\right)}^{2}}(r-r^{*})+\frac{\alpha \beta \delta }{{\left(\beta -\delta r^{*}\right)}^{3}}{\left(r-r^{*}\right)}^{2}+\text{higher order terms}$$

Neglecting the contribution of higher order terms and substituting *r* * =0 to (28), we get

(29) $$\frac{\alpha r}{\left(\beta -\delta r\right)}=\frac{\alpha }{\beta }r+\frac{\alpha \delta }{\beta^{2}}r^{2}$$

As a remark, for r^*^ ≠ 0, we can always perform a change of variables to ensure that the equilibrium is zero.

**Fig. 6:**
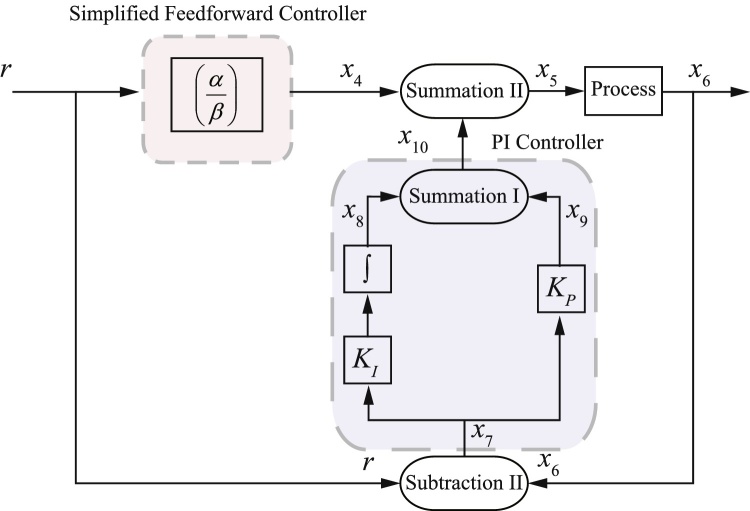
Block diagram configuration with simplified feedforward controller.

With this approximation, we have reduced the inverse-feedforward controller to requiring two gain operators, one summation operator and one polynomial operator. Substituting the relevant values of *α*, *β* and *δ*, which are related to the process parameters (i.e. *k*_*r*1_, *k*_*r*2_ and *k*_*r*3_), we obtain *u* ≈ 0.029*r* + 0.000002*r*^2^. Given that the coefficient of the second term is very small, our final approximation of the inverse-feedforward controller is then given by *u* ≈ 0.029*r*. Hence, we have further reduced the complexity of the inverse-feedforward controller to requiring only one gain operator. With that, the block diagram configuration of the resulting simplified feedforward controller is shown in Fig. 6.

Using the same variables as in the full feedforward controller case, the associated abstract chemical reaction for the simplified feedforward controller is given by

[Simplified feedforward controller:]

(30) $$\begin{array}{c} r\overset{\gamma_{G}\Gamma }{\to }r+x_{4}\\ x_{4}\overset{\gamma_{G}}{\to }\emptyset \end{array}$$

where *γ*_*G*_ is the gain reaction rate and Γ = *α*/*β*. The associated ODE is given by

(31) $$\frac{dx_{4}}{dt}=\gamma_{G}(\Gamma r-x_{4})$$

**Fig. 7:**
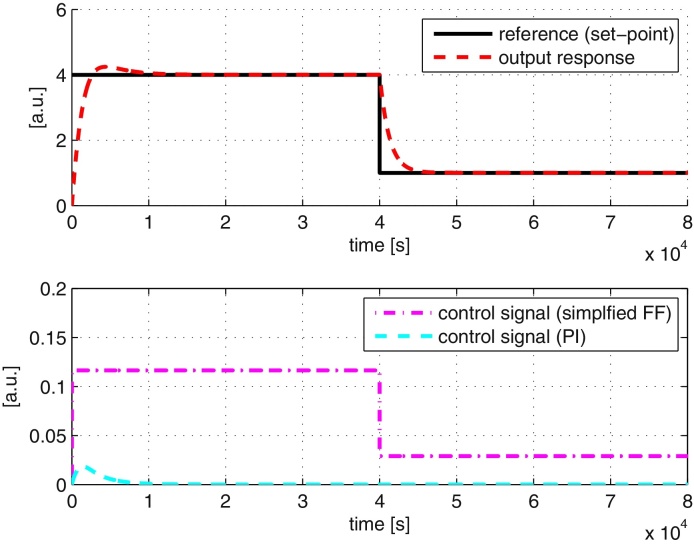
System responses to the tracking controller using simplified feedforward controller. Top: output, control and reference signals. Bottom: control signals from simplified feedforward (FF) and PI controllers.

We repeat the reference tracking simulations as in the case of a full feedforward controller and the results are shown in Fig. 7. The results show that the performance of the simplified feedforward controller is similar to the case of a full feedforward controller. However, implementation of the simplified feedforward controller requires only 18 chemical reactions, a reduction of 31% and 50% from the full feedforward controller and framework proposed in Oishi and Klavins (2011) respectively.

**Fig. 8:**
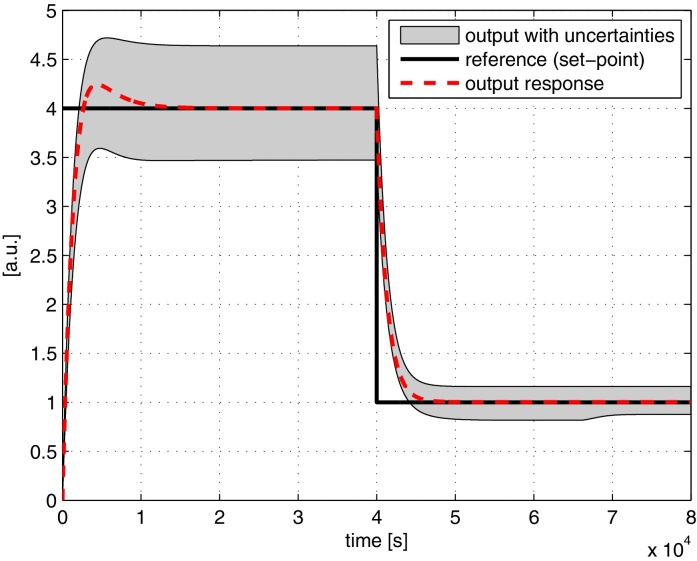
Robustness analysis of the tracking controller using the simplified feedforward controller.

We repeat the robustness analysis with the parameters $\phi \in \{\gamma_{G,i},\gamma_{G_{K_{P}},i},\gamma_{Sm_{I},j},\gamma_{Sb_{II},k}$, $\gamma_{Sm_{II},j},K_{P},K_{I},k_{r1},k_{r2},k_{r3}\}$, where *i* ∈ {1, 2}, *j* ∈ {1, 2, 3} and *k* ∈ {1, 2, 3, 4, 5}. The result is shown in Fig. 8. Again, we observe similar performance compared to the full feedforward controller. In fact the envelope of possible responses for the simplified feedforward controller is smaller compared to the full feedforward controller, since the number of uncertain parameters is reduced. The sensitivity analysis is also repeated and the results are shown in Table 3.

As in the case of the full feedforward controller, the most sensitive parameters are associated with the summation and subtraction operators as well as the gain of the simplified feedforward controller.

### 4.5 Design considerations and limitations for different biochemical processes

**Fig. 9:**
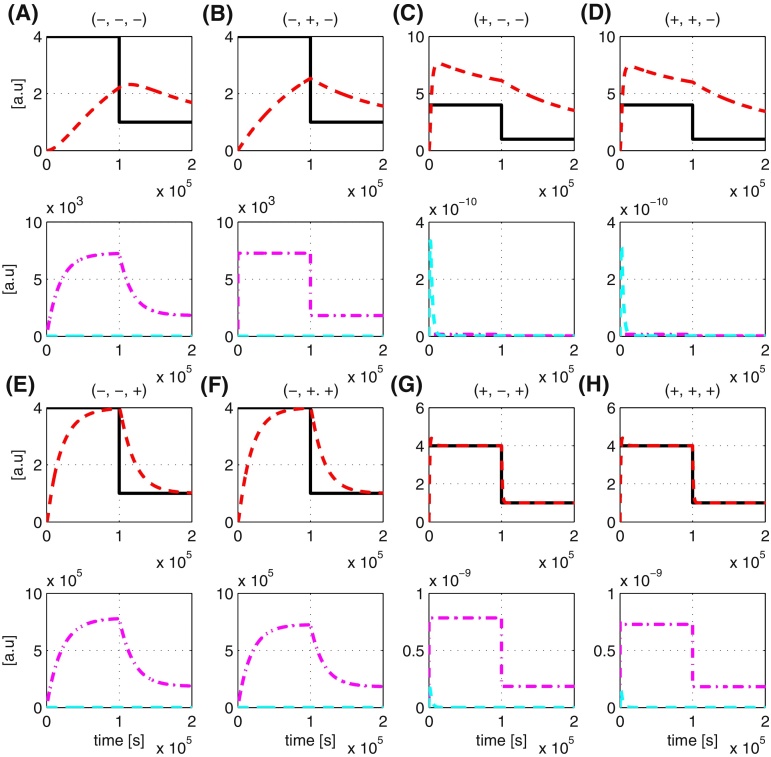
Effect of varying process parameters on reference tracking using inverse-feedforward controller. The notation ‘+’ and ‘−’ denotes respectively the maximum and minimum values of the process parameter. Red line: output response. Black line: reference (set-point). Magenta line: control signal from inverse-feedforward controller. Cyan line: control signal from PI controller. (For interpretation of the references to colour in this figure legend, the reader is referred to the web version of the article.)

**Fig. 10:**
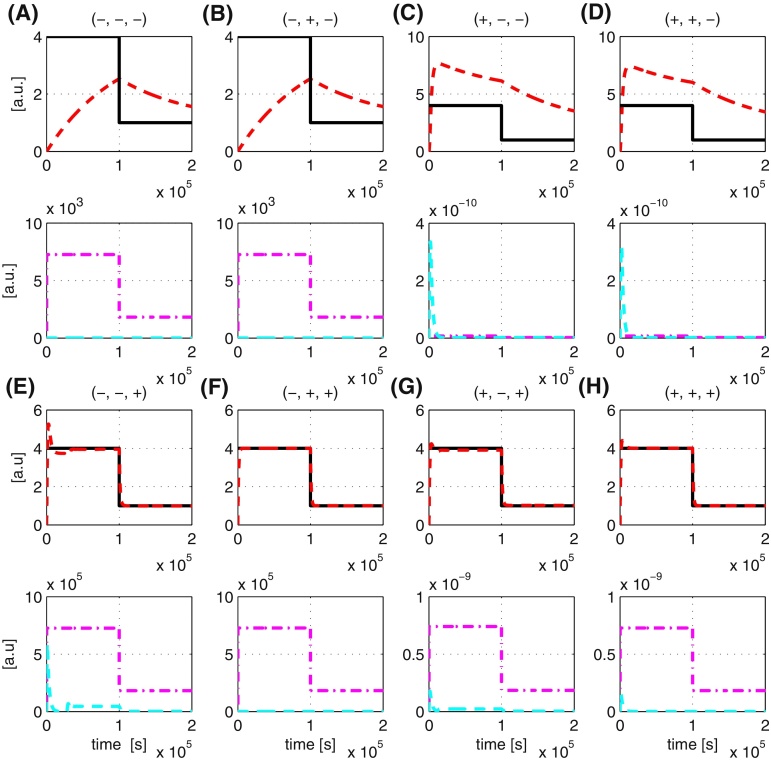
Effect of varying process parameters on reference tracking using simplified feedforward controller. The notation ‘+’ and ‘−’ denotes respectively the maximum and minimum values of the process parameter. Red line: output response. Black line: reference (set-point). Magenta line: control signal from simplified feedforward controller. Cyan line: control signal from PI controller. (For interpretation of the references to color in this figure legend, the reader is referred to the web version of the article.)

The dynamics of the enzymatic reaction process depend on *k*_*r*1_, *k*_*r*2_ and *k*_*r*3_. From the experimental biology literature, it is clear that these three parameters can vary over several orders of magnitude - depending on the particular biochemical process in question values of *k*_*r*1_, *k*_*r*2_ and *k*_*r*3_ in the literature range from 10^−9^ to 10^6^, 10^−2^ to 10^2^ and 10^−5^ to 10^−3^ respectively (see e.g. Faeder et al., 2003, Hatakeyama et al., 2003, Charusanti et al., 2004). Such large variations in the process parameters can clearly significantly impact the design of the tracking controller and need to be taken into account in the design process. Here, we investigate the effect of each of these process parameters on the performance of the tracking controller using both the full and simplified feedforward controllers. We retain all the original parameters apart from the process parameters, which we will vary in our analysis. For each process parameter, we use the minimum and maximum values given above and consider their effect independently and jointly. Table 4 summarises the findings and the simulation results are shown in Fig. 9, Fig. 10.

In Fig. 9, Fig. 10, the signs (+) and (−) represent respectively the maximum and minimum values of *k*_*r*1_ to *k*_*r*3_ of the process. For example, the notation (+,−,−) means that we are analysing the scenario where *k*_*r*1_ takes the maximum value of its range, i.e. 1 × 10^6^, and *k*_*r*2_ and *k*_*r*3_ take the minimum value of their respective range, i.e. 1 × 10^−2^ and 1 × 10^−5^. Likewise, the notation (+,+,−) represents the analysis of the scenario where *k*_*r*1_ and *k*_*r*2_ take their maximum value of their respective range and *k*_*r*3_ is at the minimum value of its range. In each of the subfigures ((A)–(H)), the top row represents the reference tracking capability of the output and the bottom row represents the control action given by both the feedforward and feedback control.

The obtained results show that the tracking controller using both full and simplified feedforward controller has difficulty in tracking reference inputs properly if the degradation term of the process *k*_*r*3_ is very small (∼10^−5^). As shown in both Fig. 9, Fig. 10(A)–(D), the control action of PI (cyan line) stays at zero most of the time indicating that the subtraction is not working properly due to *r* < *y*. Now, with a small value of *k*_*r*3_, *y* will degrade slowly, meaning *r* < *y* for a longer time, hence the poor tracking (red line). On the other hand, if *k*_*r*3_ is large (∼10^−3^), the time for which *r* < *y* is short as *y* degrades much faster, thus leading to accurate tracking as shown in both Fig. 9, Fig. 10(E)–(H).

For very small values of *k*_*r*1_, to achieve good reference tracking requires the reaction rates in the inverse-feedforward controller to be increased. For implementation using DNA-based chemistry, the ability to increase reaction rates could potentially be constrained by the physical binding property of the DNA, thus this point has to be taken into account when adjusting those reaction rates. For a large value of *k*_*r*1_, good reference tracking can be achieved by decreasing the PI controller gains *K*_*P*_ and *K*_*I*_.

On the other hand, when the simplified feedforward controller is used, a good reference tracking can be achieved by just adjusting the PI controller gain. Finally, in the case when the value of *k*_*r*1_ is small and the values of *k*_*r*2_ and *k*_*r*3_ are large, no adjustment is required to the controller parameters. In Fig. 9, Fig. 10(E) and (F), we note that despite the tracking controller being able to track the reference signal properly, potentially large control signals are required, whose feasibility needs to be checked during the design process with experimentalists.

### 4.6 Retroactivity

Here, we investigate how retroactivity affects the overall performance of the tracking controllers. In previous sections, we have assumed perfect modularity of the different elements in the closed-loop feedback control system shown in Fig. 2, Fig. 6. In other words, the dynamic responses are not affected by the interconnection of the components. While this assumption is widely made in the analysis of chemical reaction based systems, recent work (see e.g. Del Vechhio et al., 2008) has shown that such an assumption does not hold for many biomolecular feedback systems. As shown in Del Vechhio et al. (2008), the occurrence of different modules sharing the same molecular species is common, and this sharing of species can affect the overall dynamics of the processes upon their interconnection. To quantify the way that interconnection of two modules alters their dynamics with respect to their behaviour in isolation, the concept of retroactivity has been introduced (Del Vecchio and Jayanthi, 2008, Jayanthi et al., 2013). For the process considered here, it should be noted that no retroactivity effects are present due to the interconnection of modules involving unimolecular reactions. For example, for the closed-loop feedback control systems in Fig. 2, Fig. 6, retroactivity does not affect the interconnection of the module Subtraction II with the PI controller.

On the other hand, an interconnection of two modules, where one module comprises unimolecular reactions while the other module comprises bimolecular reactions, will feature a unidirectional retroactivity. Again, using an example in the context of the closed-loop feedback control system shown in both Fig. 2, Fig. 6, retroactivity affects the interconnection of the module Summation II with the process (see (8), (21)). To take into account the effect of retroactivity, the ODE representation of Summation II must consider the chemical reactions downstream of the process (see (25)). Thus the ODE representation of the Summation II module including the effect of retroactivity is given as follows:

(32) $$\frac{dx_{5}}{dt}=\gamma_{Sm_{II}}(x_{4}+x_{10}-x_{5})-\underset{\mathrm{retroactivity}}{\underset{︸}{k_{r1}x_{5}x_{e}}}$$

**Fig. 11:**
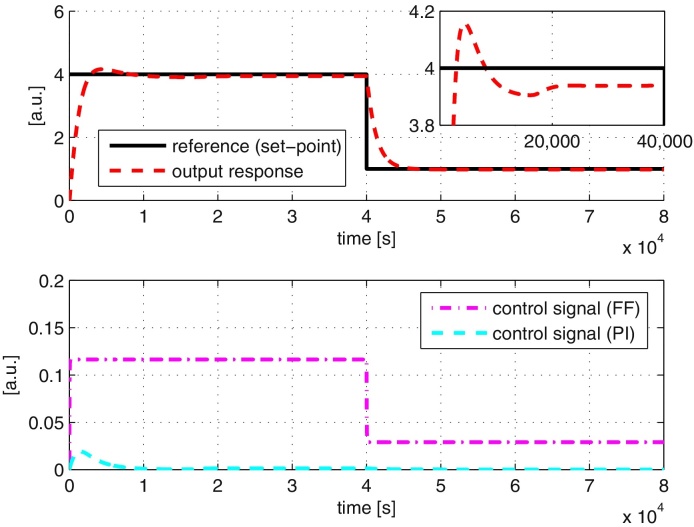
System responses with retroactivity using inverse-feedforward controller. Top: output and reference signals. Bottom: control signals from inverse-feedforward (FF) and PI controllers.

**Fig. 12:**
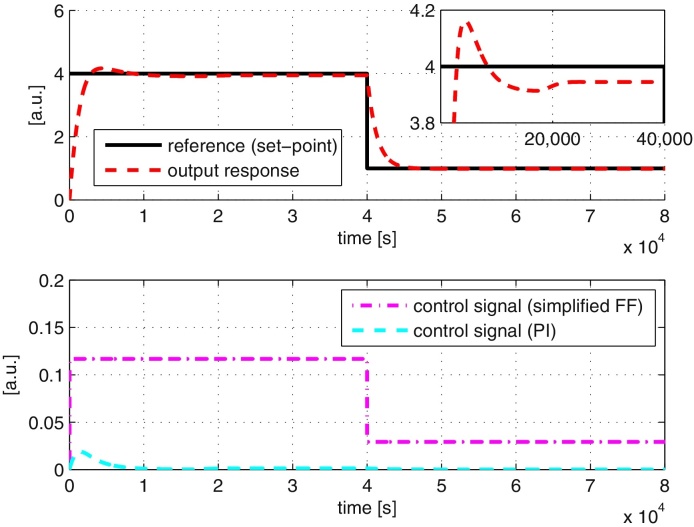
System responses to retroactivity using simplified feedforward controller. Top: output, control and reference signals. Bottom: control signals from simplified feedforward (FF) and PI controllers.

We repeat the simulations in Section 4.2 using both the full and simplified feedforward controller taking into account the effects of retroactivity, and the results are shown in Fig. 11, Fig. 12. The results show that retroactivity does not significantly affect the performance of the overall closed-loop system other than to introduce a very small steady state error. The reason for this steady state error can be explained as the following. The retroactivity affects the input to the process, *x*_5_. At the same time, *x*_5_ is also the resulting control signal from both the feedforward and PI controllers. As both the controllers do not have information regarding this error resulting from retroactivity, these controllers are unable to provide the correct control signal to react to the effect of retroactivity, thus leading to the observed steady state error. Nevertheless, our results show that the performance of the tracking controller is not significantly affected by retroactivity, allowing us to undertake designs based on the assumption of modularity (as is the case with standard control applications).

### 4.7 Implementation of CRN via DNA strand displacement chemistry

Finally, we briefly provide an overview of the way in which the type of designs presented in this paper may be implemented experimentally. Recently, there have been a number of studies that described how synthetic circuits composed of abstract chemical reactions may be readily implemented using DNA-based chemistry (see e.g. Seelig et al., 2006, Soloveichik et al., 2010, Qian and Winfree, 2011, Chen et al., 2013). In Chen et al. (2013), the authors highlighted that chemical reactions can serve as a programming language for the design of DNA-based chemistry synthetic circuits. Therefore, circuit components synthesised using DNA that can be expressed mathematically can be derived from biologically synthesised plasmids. Consequently, in principle this enables the in vitro implementation of those circuits. The advantage of utilising DNA-based chemistry in the design lies in the ease of implementation, as the design depends on the choice of relevant sequences following the standard Watson-Crick pairing (i.e. adenine-thymine and guanine-cytosine or A-T and G-C). As a result, design frameworks and software tools that allow synthetic biomolecular circuitry described using CRN's to be readily implemented using DNA strand displacement (DSD) chemistry have been developed in recent years (see e.g. Seelig et al., 2006, Soloveichik et al., 2010, Qian and Winfree, 2011, Chen et al., 2013).

In particular, it has been shown in Soloveichik et al. (2010) that unimolecular (one reactant at the LHS of the chemical reaction) and bimolecular (two reactants at the LHS of the chemical reaction) chemical reactions can be compiled in DSD chemistry to accomplish the intended behaviour of their designed biomolecular circuit. All designs presented in this work are all made up of either unimolecular or bimolecular chemical reaction, making this framework suitable for the designed controller to be implemented experimentally. Here, we briefly describe their proposed framework and refer interested readers to Soloveichik et al. (2010) for details.

Consider the following bimolecular DSD reaction,

(33) $$X+Q\overset{k_{b}}{\underset{k_{ub}}{⇌}}Y+W$$

where *k*_*b*_ is the binding reaction rate and *k*_*ub*_ is the unbinding reaction rate of the DNA strand. The reaction commences when the invader strand *Q* binds in a standard Watson–Crick complementary manner to the toe-hold domain of strand *X*. As the binding occurs, portions of the strand of *X* are displaced whereby this separation results in the product *Y* and waste *W*. This partially double stranded product *Y* can then bind with to the toe-hold domain of other DNA strands and complexes for subsequent reactions. The rate of the overall reaction can be controlled by varying the binding and unbinding rates, *k*_*b*_ and *k*_*ub*_. Quite often, different DNA strands do not interact directly with one another, and hence auxiliary species with sufficiently large amount are required to facilitate their interaction. With the inclusion of auxiliary species, the DSD implementation for the respective unimolecular (*X* → *Y* + *Z*) and bimolecular (*X* + *Y* → *Z*) reactions are given by,

(34) $$\begin{array}{c} X+G\overset{q}{\to }O\\ O+T\overset{q_{max}}{\to }Y+Z \end{array}$$

and

(35) $$\begin{array}{c} X+L\overset{q}{\underset{q_{max}}{⇌}}H+B\\ Y+H\overset{q_{max}}{\to }O\\ O+T\overset{q_{max}}{\to }Z \end{array}$$

where *G*, *O*, *T*, *L*, *H* and *B* are the auxiliary species with appropriate initial concentrations *C*_*max*_. The partial strand displacement rate is given by *q* = *k*_*b*_/*C*_*max*_ and *q*_*max*_ is the maximum strand displacement rate.

Since the abstract chemical reactions describing the tracking controller presented here are made up exclusively of unimolecular and bimolecular reactions, the framework introduced in Soloveichik et al. (2010) enables our design to be implemented via DNA chemistry. Nevertheless, since the introduction of auxiliary species further increases the number of reaction needed for DSD implementations, it is imperative that circuit designs utilise as few reactions as possible. Hence, our results showing the feasibility of a radically simplified inverse-feedforward tracking controller offer strong potential for future wet lab implementations of embedded biomolecular control systems.

## 5 Conclusions

In this paper, we have shown for the first time how a controller architecture for implementing reference tracking, based on the use of an inverse-feedforward controller, can be adapted to the specific context of embedded biomolecular feedback systems. Our proposed approach circumvents a fundamental limitation of CRN-based systems from the point of view of tracking control, i.e. the inability of standard CRNs to implement a two-sided subtraction operator. The only currently available solution to this problem proposed by Oishi and Klavins (2011) requires the adoption of a non-standard modelling and design framework that represents signals as the difference in the concentration of two chemical species, an approach that significantly increases the complexity of the design process and doubles the total number of chemical reactions needed to implement a given circuit.

The complexity of the control problem considered here is significantly higher than in previous studies – almost all previous studies consider either a static or a very simple first order process that avoids the possibility of the reference value being larger than the output value (Cosentino et al., 2016) or employing control strategies that can reduce the duration of the reference value being smaller than the output value (Briat et al., 2016) or consider only a very simple static process with no dynamics (Yordanov et al., 2014). Using the existing standard realisation of one-sided subtraction, our approach of using inverse feedforward combined with feedback control produces highly accurate and robust reference tracking. By exploiting the biochemical structure of the feedforward controller, we are able to reduce its complexity using a Taylor series approximation. The resulting simplified feedforward control not only achieves similar performance to the full feedforward controller, but also utilises far fewer chemical reactions. The reduction in the number of chemical reactions is important, as it will significantly facilitate the experimental implementation of the proposed design in DNA-based chemistry either in vitro or in vivo.

## Conflict of interest

The authors declare that they have no competing interests.

## Figures and Tables

**Table 1:** Parameters used in the closed-loop feedback control system.

Parameters	Values
*Process*	
*k*_*r*1_	0.005
*k*_*r*2_	1.6
*k*_*r*3_	0.0008
*x*_*T*_	5.5

*Inverse-feedforward controller*	
$\gamma_{G_{\alpha }}$	1.0
$\gamma_{G_{\delta }}$	1.0
$\gamma_{G_{Sb_{I}}}$	1.0
$\gamma_{G_{D}}$	1.0

*PI controller*	
$\gamma_{G_{K_{P}}}$	1.0
*K*_*P*_	0.02
*K*_*I*_	2.5 × 10^−8^
$\gamma_{Sm_{I}}$	0.0004

*Summation II and Subtraction II*	
$\gamma_{{\mathrm{Sm}}_{\mathrm{II}}}$	1.0
$\gamma_{Sb_{\mathrm{II}}}$	3.0

**Table 2:** Parameter sensitivity analysis of the tracking controller. The maximum percentage relative steady state error has two rows for each parameter, where the upper and bottom rows denote Relative *e*_*ss*,*U*_ and Relative *e*_*ss*,*D*_ respectively.

Parameters	Rel. *e*_*ss*_ (%)	Parameters	Rel. *e*_*ss*_ (%)	Parameters	Rel. *e*_*ss*_ (%)
*Subtraction II*		*Inverse-feedforward controller*		*Summation II*	
$\gamma_{Sb_{II},1}$	29.58	$\gamma_{G_{\alpha },1}$	50.00	$\gamma_{Sm_{II},1}$	50.00
	31.97		50.00		50.00
$\gamma_{Sb_{II},2}$	20.95	$\gamma_{G_{\alpha },2}$	50.00	$\gamma_{Sm_{II},2}$	0.25
	23.08		50.00		0.99
$\gamma_{Sb_{II},3}$	0.25	$\gamma_{G_{\delta },1}$	0.25	$\gamma_{Sm_{II},3}$	50.00
	0.99		0.00		50.00
$\gamma_{Sb_{II},4}$	20.95	$\gamma_{G_{\delta },2}$	0.25	*Process*	
	23.08		0.00	*k*_*r*1_	0.25
$\gamma_{Sb_{II},5}$	20.95	$\gamma_{Sb_{I},1}$	50.00		0.99
	23.08		50.00	*k*_*r*2_	0.00
*PI controller*		$\gamma_{Sb_{I},2}$	0.25		0.00
*K*_*I*_	0.25		0.00	*k*_*r*3_	0.25
	0.99	$\gamma_{Sb_{I},3}$	50.00		0.99
*K*_*P*_	0.00		50.00		
	0.00	$\gamma_{Sb_{I},4}$	0.25		
$\gamma_{Sm_{I},1}$	0.25		0.00		
	0.99	$\gamma_{Sb_{I},5}$	0.25		
$\gamma_{Sm_{I},2}$	0.00		0.00		
	0.00	$\gamma_{G_{D},1}$	50.00		
$\gamma_{Sm_{I},3}$	0.25		50.00		
	0.99	$\gamma_{G_{D},2}$	50.00		
$\gamma_{G_{K_{P}},1}$	0.00		50.00		
	0.00				
$\gamma_{G_{K_{P}},2}$	0.00				
	0.00				

**Table 3:** Parameter sensitivity analysis of the tracking controller using the simplified feedforward controller. The maximum percentage of relative steady state error has two rows, where the upper and bottom rows denote Relative *e*_*ss*,*U*_ and Relative *e*_*ss*,*D*_ respectively.

Parameters	Rel. *e*_*ss*_ (%)	Parameters	Rel. *e*_*ss*_ (%)
*Subtraction II*		*Simplified feedforward controller*	
$\gamma_{Sb_{II},1}$	29.58	*γ*_*G*,1_	50.12
	31.97		50.25
$\gamma_{Sb_{II},2}$	21.10	*γ*_*G*,2_	50.12
	23.08		50.25
$\gamma_{Sb_{II},3}$	0.50	*Summation II*	
	0.99	$\gamma_{Sm_{II},1}$	50.12
$\gamma_{Sb_{II},4}$	21.10		50.25
	23.08	$\gamma_{Sm_{II},2}$	0.50
$\gamma_{Sb_{II},5}$	21.10		0.99
	23.08	$\gamma_{Sm_{II},1}$	50.12
*PI controller*			50.25
*K*_*I*_	0.50	*Process*	
	0.99	*k*_*r*1_	0.74
*K*_*P*_	0.50		0.99
	0.99	*k*_*r*2_	0.74
$\gamma_{Sm_{I},1}$	0.50		0.99
	0.99	*k*_*r*3_	0.74
$\gamma_{Sm_{I},2}$	0.50		0.99
	0.99		
$\gamma_{Sm_{I},3}$	0.50		
	0.99		
$\gamma_{G_{K_{P}},1}$	0.50		
	0.99		
$\gamma_{G_{K_{P}},2}$	0.50		
	0.99		

**Table 4:** Effect of process parameters on reference tracking capability and remarks on the control design guidelines. For the reference tracking capability, Y denotes Yes and N denotes No.

Reference tracking capability (Y/N)	*k*_*r*1_	*k*_*r*2_	*k*_*r*3_	Design remarks
*Inverse-feedforward controller*				
N	1 × 10^−9^	1 × 10^−2^	1 × 10^−5^	
N	1 × 10^−9^	1 × 10^2^	1 × 10^−5^	
N	1 × 10^6^	1 × 10^−2^	1 × 10^−5^	
N	1 × 10^6^	1 × 10^2^	1 × 10^−5^	
Y	1 × 10^−9^	1 × 10^−2^	1 × 10^−3^	Increase reaction rates *γ*
Y	1 × 10^−9^	1 × 10^2^	1 × 10^−3^	Increase reaction rates *γ*
Y	1 × 10^6^	1 × 10^−2^	1 × 10^−3^	Decrease *K*_*P*_ and *K*_*I*_
Y	1 × 10^6^	1 × 10^2^	1 × 10^−3^	Decrease *K*_*P*_ and *K*_*I*_

*Simplified feedforward controller*				
N	1 × 10^−9^	1 × 10^−2^	1 × 10^−5^	
N	1 × 10^−9^	1 × 10^2^	1 × 10^−5^	
N	1 × 10^6^	1 × 10^−2^	1 × 10^−5^	
N	1 × 10^6^	1 × 10^2^	1 × 10^−5^	
Y	1 × 10^−9^	1 × 10^−2^	1 × 10^−3^	Increase *K*_*P*_ and *K*_*I*_
Y	1 × 10^−9^	1 × 10^2^	1 × 10^−3^	No change to existing parameters
Y	1 × 10^6^	1 × 10^−2^	1 × 10^−3^	Decrease *K*_*P*_ and *K*_*I*_
Y	1 × 10^6^	1 × 10^2^	1 × 10^−3^	Decrease *K*_*P*_ and *K*_*I*_

## References

[bib0005] Baldea M., El-Farra N.H., Ydstie B.E. (2013). Dynamics and control of chemical process networks: integrating physics, communication and computation. Comput. Chem. Eng..

[bib0010] Bilotta M., Cosentino C., Bates D.G., Salerno L., Amato F. (2015). Retroactivity analysis of a chemical reaction network module for the subtraction of molecular fluxes. Proceedings of IEEE Conference on Engineering in Medicine and Biology Society.

[bib0015] Bilotta M., Cosentino C., Merola A., Bates D.G., Amato F. (2016). Zero-retroactivity subtraction module for embedded feedback control of chemical reaction networks. Proceedings of IFAC Conference on Foundations of Systems Biology in Engineering.

[bib0020] Briat C., Zechner C., Khammash M. (2016). Design of a synthetic integral feedback circuit: dynamic analysis and DNA implementation. ACS Synth. Biol..

[bib0025] Buisman H.J., ten Eikelder H.M.M., Hilbers P.A.J., Liekens A.M.L. (2009). Computing algebraic functions with biochemical reaction networks. Artif. Life.

[bib0030] Charusanti P., Hu X., Chen L., Neuhauser D., DiStefano J.J. (2004). A mathematical model of BCR-ABL autophosphorylation, signaling through the CRKL pathway and Gleevec dynamics in chronic myeloid leukemia. Discrete Contin. Dyn. Syst. Ser. B.

[bib0035] Chen Y.J., Dalchau N., Srinivas N., Phillips A., Cardelli L., Soloveichik D., Seelig G. (2013). Programmable chemical controllers make from DNA. Nat. Nanotechnol..

[bib0040] Cosentino C., Ambrosino R., Ariola M., Bilotta M., Pironti A., Amato F. (2016). On the realization of an embedded subtractor module for the control of chemical reaction network. IEEE Trans. Autom. Control.

[bib0045] Cosentino C., Bilotta M., Merola A., Amato F. (2013). A synthetic biology approach to the realization of embedded feedback controllers for chemical reaction networks. Proceedings of IEEE Conference on Bioinformatics and Bioengineering.

[bib0050] Del Vecchio D., Jayanthi S. (2008). Retroactivity attenuation in transcriptional networks: design and analysis of an insulation device. Proceedings of IEEE Conference on Decision and Control.

[bib0055] Del Vechhio D., Ninfa A., Sontag E. (2008). Modular cell biology: retroactivity and insulation. Mol. Syst. Biol..

[bib0060] Devasia S. (2002). Should model-based inverse inputs be used as feedforward under plant uncertainty?. IEEE Trans. Autom. Control.

[bib0065] Faeder J.R., Hlavacek W.S., Reischl I., Blinov M.L., Metzger H., Redondo A., Wofsy C., Goldstein B. (2003). Investigation of early events in fc*ϵ*ri-mediated signaling using a detailed mathematical model. J. Immunol..

[bib0070] Feinberg M. (1986). Chemical reaction network structure and the stability of complex isothermal reactors – I. The deficiency zero and deficiency one theorems. Chem. Eng. Sci..

[bib0075] Feinberg M. (1988). Chemical reaction network structure and the stability of complex isothermal reactors – II. Multiple steady states for network deficiency one. Chem. Eng. Sci..

[bib0080] Franklin G.F., Powell J.D., Emami-Naeini A. (2014). Feedback Control of Dynamic Systems.

[bib0085] Galazzo J.L., Bailey J.E. (1990). Fermentation pathway kinetics and metabolic flux control in suspended and immobilized *S. cerevisiae*. Enzyme Microb. Technol..

[bib0090] Gershon E., Shaked U. (2008). *H*_∞_ feedback-control theory in biochemical systems. Int. J. Robust Nonlinear Control.

[bib0095] Harris A.W.K., Dolan J., Kelly C., Anderson J., Papachristodoulou A. (2015). Designing genetic feedback controllers. IEEE Trans. Biomed. Circuits Syst..

[bib0100] Hatakeyama M., Kimura S., Naka T., Kawasaki T., Yumoto N., Ichikawa M., Kim J.H., Saito K., Saeki M., Shirouzu M., Yokoyama S., Konagaya A. (2003). A computational model on the modulation of mitogen-activated protein kinase (MAPK) and Akt pathways in heregulin-induced ErbB signalling. Biochem. J..

[bib0105] Heinrich R., Neel B.G., Rapoport T.A. (2002). Mathematical models of protein kinase signal transduction. Mol. Cell.

[bib0110] Henson M.A. (2003). Dynamic modeling and control of yeast cell populations in continuous biochemical reactors. Comput. Chem. Eng..

[bib0115] Hsiao V., de los Santos E.L.C., Whitaker W.R., Dueber J.E., Murray R.M. (2015). Design and implementation of a biomolecular concentration tracker. ACS Synth. Biol..

[bib0120] Jayanthi S., Nilgiriwala K.S., Del Vecchio D. (2013). Retroactivity controls the temporal dynamics of gene transcription. ACS Synth. Biol..

[bib0125] Lin H.Y., Chen J.Z., Li H.Y., Yang C.N. (2015). A simple three-input DNA-based system works as a full-subtractor. Sci. Rep..

[bib0130] Menon P.P., Postlethwaite I., Bennani S., Marcos A., Bates D.G. (2009). Robustness analysis of a reusable launch vehicle flight control law. Control Eng. Pract..

[bib0135] Ogata K. (2010). Modern Control Engineering.

[bib0140] Oishi K., Klavins E. (2011). Biomolecular implementation of linear I/O systems. IET Syst. Biol..

[bib0145] Qian L., Winfree E. (2011). A simple DNA gate motif for synthesizing large-scale circuits. J. R. Soc. Interface.

[bib0150] Rivera D., Morari M., Skogestad S. (1986). Internal model control: PID controller design. Ind. Eng. Chem. Process Des. Dev..

[bib0155] Salehi S.A., Parhi K.K., Riedel M.D. (2016). Chemical reaction networks for computing polynomials. ACS Synth. Biol..

[bib0160] Seelig G., Soloveichik D., Zhang D.Y., Winfree E. (2006). Enzyme-free nucleic acid logic circuits. Science.

[bib0165] Segel I.R. (1975). Enzyme Kinetics – Behaviour and Analysis of Rapid Equilibrium and Steady-state Enzymes Systems.

[bib0170] Soloveichik D., Seelig G., Winfree E. (2010). DNA as a universal substrate for chemical kinetics. Proc. Natl. Acad. Sci. U. S. A..

[bib0175] Song T., Garg S., Mokhtar R., Bui H., Reif J. (2016). Analog computation by DNA strand displacement circuits. ACS Synth. Biol..

[bib0180] Taylor D.J., Green N.P.O., Stout G.W., Soper R. (2008). Biological Science.

[bib0185] Vidyasagar M. (1998). Statistical learning theory and randomised algorithm for control. IEEE Control Syst..

[bib0190] Williams P.S. (2001). A Monte Carlo dispersion analysis of the x-33 simulation software. Proceedings of AIAA Conference on Guidance, Navigation and Control.

[bib0195] Xu S., Li H., Miao Y., Liu Y., Wang E. (2013). Implementation of half adder and half subtractor with a simple and universal DNA-based platform. NPG Asia Mater..

[bib0200] Yordanov B., Kim J., Petersen R.L., Shudy A., Kulkarni V.V., Phillips A. (2014). Computational design of nucleic acid feedback control circuits. ACS Synth. Biol..

